# Transient *in utero *disruption of Cystic Fibrosis Transmembrane Conductance Regulator causes phenotypic changes in Alveolar Type II cells in adult rats

**DOI:** 10.1186/1471-2121-10-24

**Published:** 2009-03-31

**Authors:** Ashraf Gad, Delon L Callender, Erin Killeen, Joseph Hudak, Malgosia A Dlugosz, Janet E Larson, J Craig Cohen, Avinash Chander

**Affiliations:** 1 The Brady Laboratory, Department of Pediatrics, Division of Neonatology, Stony Brook University Medical Center, Stony Brook, NY 11794, USA

## Abstract

**Background:**

Mechanicosensory mechanisms regulate cell differentiation during lung organogenesis. We have previously demonstrated that cystic fibrosis transmembrane conductance regulator (CFTR) was integral to stretch-induced growth and development and that transient expression of antisense-CFTR (ASCFTR) had negative effects on lung structure and function. In this study, we examined adult alveolar type II (ATII) cell phenotype after transient knock down of CFTR by adenovirus-directed *in utero *expression of ASCFTR in the fetal lung.

**Results:**

In comparison to (reporter gene-treated) Controls, ASCFTR-treated adult rat lungs showed elevated phosphatidylcholine (PC) levels in the large but not in the small aggregates of alveolar surfactant. The lung mRNA levels for SP-A and SP-B were lower in the ASCFTR rats. The basal PC secretion in ATII cells was similar in the two groups. However, compared to Control ATII cells, the cells in ASCFTR group showed higher PC secretion with ATP or phorbol myristate acetate. The cell PC pool was also larger in the ASCFTR group. Thus, the increased surfactant secretion in ATII cells could cause higher PC levels in large aggregates of surfactant. In freshly isolated ATII cells, the expression of surfactant proteins was unchanged, suggesting that the lungs of ASCFTR rats contained fewer ATII cells. Gene array analysis of RNA of freshly isolated ATII cells from these lungs showed altered expression of several genes including elevated expression of two calcium-related genes, Ca^2+^-ATPase and calcium-calmodulin kinase kinase1 (CaMkk1), which was confirmed by real-time PCR. Western blot analysis showed increased expression of calmodulin kinase I, which is activated following phosphorylation by CaMkk1. Although increased expression of calcium regulating genes would argue in favor of Ca^2+^-dependent mechanisms increasing surfactant secretion, we cannot exclude contribution of alternate mechanisms because of other phenotypic changes in ATII cells of the ASCFTR group.

**Conclusion:**

Developmental changes due to transient disruption of CFTR in fetal lung reflect in altered ATII cell phenotype in the adult life.

## Background

Cell development and differentiation in the fetal lung are regulated by mechanical, physiologic, and biochemical factors [1]. The markers for peripheral lung development include maturation of alveolar type II (ATII) cells as evidenced by the appearance of lamellar bodies and increased expression of surfactant phospholipids and proteins, and transformation of ATII cells into type I cells [2-4]. Previously studies have demonstrated that cystic fibrosis transmembrane conductance regulator (CFTR) mRNA and protein are expressed in adult ATII cells and that the CFTR-like chloride channel is functional [5-7]. However, its role in development of fetal ATII cells is unclear. One possible mechanism is that CFTR-mediated chloride secretion in the fetal lung epithelium can concomitantly increase fluid secretion and lung fluid volume [7,8]. Lung distention due to increased fluid secretion and fluid volume can accelerate peripheral lung development and ATII cell maturation as demonstrated in tracheal occlusion studies [9,10]. Conversely, decreased lung volume in congenital diaphragmatic hernia can result in impaired lung growth and differentiation [9,11]. Multiple studies utilizing *in utero *gene transfer and transient over-expression of CFTR have shown increased fetal lung volume, and accelerated maturation of ATII cells [11-14].

Mutations of CFTR gene have been associated with Cystic Fibrosis (CF); however, the mechanism for its direct participation in the disease pathology remains unclear [1]. CFTR is found in the epithelial cells of many organs including the lung. The crucial role of CFTR in the cellular development and cell differentiation in the lung has become somewhat clear with studies involving *in utero *gene transfer technology developed by Larson and Cohen [13,15,16]. This technique circumvents the early developmental role of CFTR and allows investigations into the role of CFTR (or any other gene) in a stage-specific manner in accessible organs. Using this technique, recent studies have shown a role of CFTR in fetal lung development because its over-expression increases mechanical stretch in the lung [12].

Pulmonary surfactant is essential for the biophysical and immunologic integrity of the lungs and for maintenance of the patency of small airways and alveoli [17-19]. Phosphatidylcholine (PC) is the major phospholipid and principal surface-active constituent in pulmonary surfactant. Four surfactant proteins – SP-A, SP-B, SP-C, and SP-D – are present, each of which plays a role in lowering of the surface tension or in the innate host-defense mechanisms in the lung. Several investigations have used differential centrifugation of the bronchoalveolar lavage (BAL) fluid for further fractionation into large aggregates (LA) and small aggregates (SA) of lung surfactant [20]. The LA fraction contains multilamellar structures, the tubular myelin, surfactant phospholipids and most of the SP-A, SP-B and SP-C proteins. It represents the newly secreted surface-active form of alveolar surfactant, which is capable of reducing the surface tension and is the precursor for the lighter SA fraction of lung surfactant and contains mostly small vesicles, surfactant phospholipids and small amounts of SP-A, SP-B and SP-C proteins [20-22].

The secretion of surfactant phospholipids in ATII cells occurs by exocytosis of lamellar bodies [23,24]. Surfactant homeostasis is under feedback regulation by surfactant proteins which increase surfactant clearance and uptake by ATII cells [23,25-27]. Different physiological, biochemical and pharmacological factors also regulate surfactant secretion through specific cell surface receptors like β-adrenergic, purinergic and endothelin receptors or by directly affecting second messenger like calcium, cAMP, and diacylglycerol [23,28,29]. Although the expression of surfactant phospholipids and proteins increases with lung and ATII cell maturation, temporal expression of each constituent may be independently regulated [30-32].

Adenovirus-mediated transfer of genes into cells has been used in several cell types including some hard to transfect cells like ATII cells. Our group has previously used this technique for *in utero *transfer of CFTR constructs and demonstrated gene-specific effects on lung development in various species [4,11-13,16,33-37]. The expression of adenovirus-mediated transferred gene was transient and lasted only 48–72 h post-transfer [13,34]. In our previous studies, we have repeatedly shown lack of inflammation in control animals that were treated with adenovirus-reporter gene constructs confirming our interpretations that the effects were due to the target gene transfer into presumably pluripotent cells in the developing lung. Our previous study employing *in utero *treatment with antisense-CFTR (ASCFTR) construct showed airway thickening with collagen deposits in the small and large airways and increased airway reactivity to acetylcholine in 100 days old rats [34].

This study was undertaken to determine if transient disruption of lung organogenesis can result in altered phenotype of ATII cells in the adult lung. Because our previous studies suggested accelerated maturation of ATII cells with transient over expression of CFTR in fetal lung [13], we postulated that a transient knock down of CFTR in fetal lung would cause delayed or erratic development and cause persistent effects in ATII cell phenotype. We now show that a similar approach utilizing transient *in utero *disruption of CFTR caused changes in surfactant protein expression and in the alveolar surfactant phospholipid pool in lungs of adult ASCFTR rats. Studies with isolated adult lung ATII cells from these animals also showed changes in surfactant secretion suggesting altered cell phenotype. The latter was also confirmed by gene array analysis of RNA from freshly isolated ATII cells, which showed altered expression of several genes including calcium regulating genes, the Ca^2+^-transporting ATPase (Atp2c2, also known as Spca2) and calcium-dependent calmodulin kinase kinase1 (CaMkk1). The latter is known to cause phosphorylation and stimulation of calcium-calmodulin kinase I (CaMKI) [38,39]. The expression levels of CaMKI, which also regulates surfactant secretion in ATII cells [40], were elevated in ATII cells from ASCFTR rats. Thus, transient *in utero *knock down of CFTR causes changes in lung development which is reflected in altered ATII cell phenotype in the adult lung.

## Results

### Studies in the lung tissue

#### Airways & Parenchymal changes

**Figure 1:**
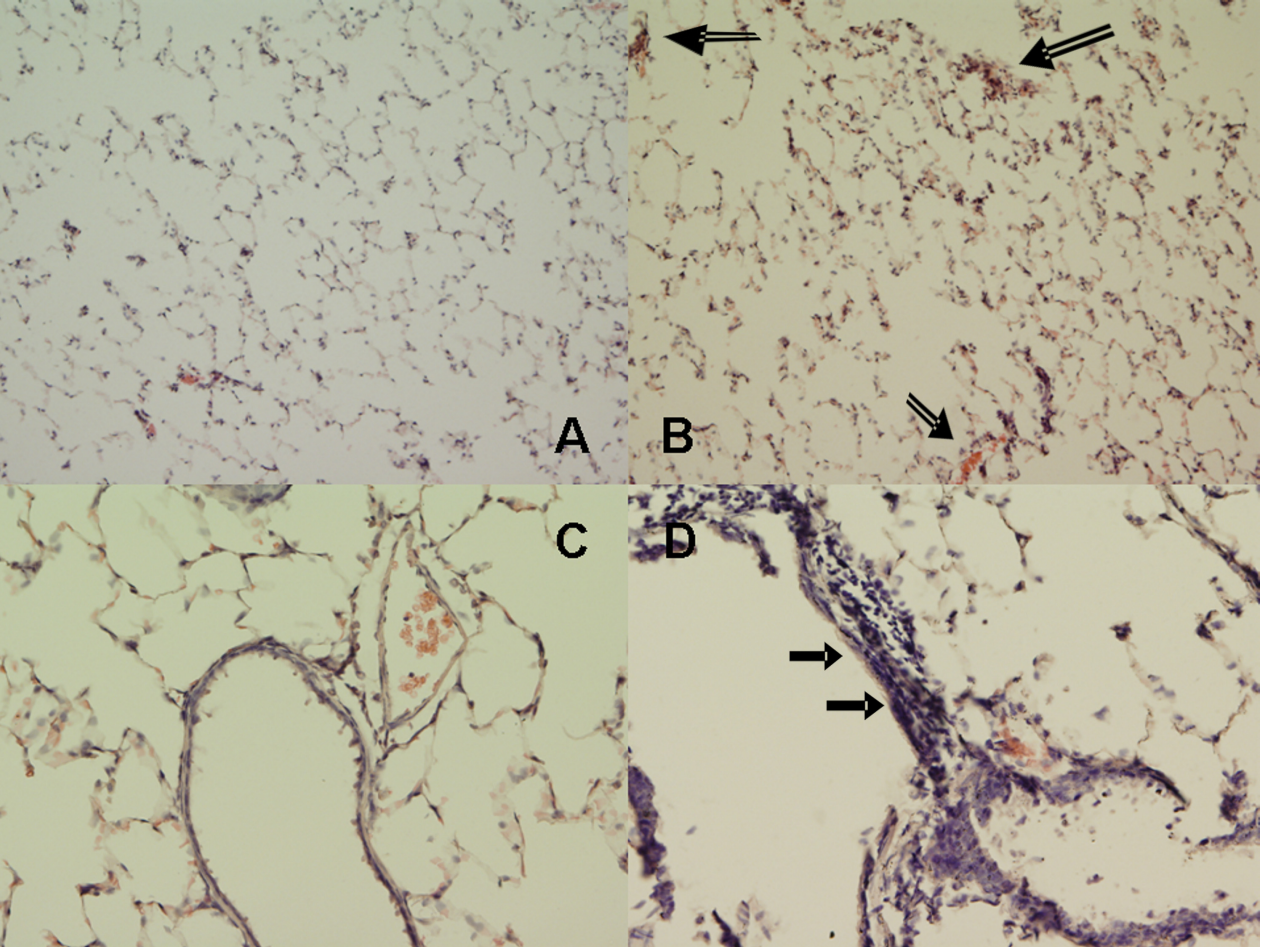
***In utero *treatment with ASCFTR causes structural changes in the lung of adult rats**. Lung sections from 3 months old rats were stained with hematoxylin and eosin and viewed by light microscopy. Frequent areas showing airway thickening and multilayered epithelia (filled arrows) were observed in the ASCFTR rat lungs **(B and D)**, while the control lungs showed normal architecture **(A and C)**. Some alveolar areas (open arrows) with cellular infiltration were also observed in the ASCFTR rats. Original magnification, **A and B **×100; **C and D **×200

The model of *in utero *gene treatment has been used in our laboratories for several investigations to determine the role of CFTR in fetal lung development. This model utilizing intra-amniotic delivery of target gene is designed for gestation periods when sufficient fluid is present in the amniotic sac for fluid injection. Although transgene expression decreases rapidly in the 30 days post-transfer [41], it is noteworthy that the CFTR knock down in these animals lasts only for 48–72 h post-transfer [13,34]. In our study, we observed structural changes in the 3-month old ASCFTR rats as evidenced by peribronchial thickening with multicellular airway epithelia suggesting thickening of airways (figures 1B and 1D) in comparison to the controls (figures 1A and 1C). A previous study from our group had noticed similar changes including interstitial lung fibrosis in 100 days old rats that were treated with ASCFTR *in utero *[34]. Lung pathology in human Cystic Fibrosis (CF) and CFTR knock down animal models is characterized by chronic inflammatory changes [36], which preclude a single causative factor for CF. Nevertheless, lung pathology and inflammation is observed in young CF patients without any overt bacterial infections [reviewed in [42]]. Previous studies from our group have indicated lung inflammation in rats that were treated with the ASCFTR constructs *in utero*. In the current study also, we observed some cell infiltrates in lung parenchyma of ASCFTR (figures 1B and 1D), but not in the Control rats (figures 1A and 1C). In the present study, we did not evaluate neutrophil infiltration and resulting inflammation in the ASCFTR animals. Thus, it is unlikely that the study animals suffered from chronic bacterial infection even though the ASCFTR animals (as well as the control animals) were maintained under normal housing conditions. Thus, this study confirming reproducibility of our model of *in utero *gene treatment with ASCFTR was not extended for additional histological studies.

#### Surfactant phospholipids

**Figure 2:**
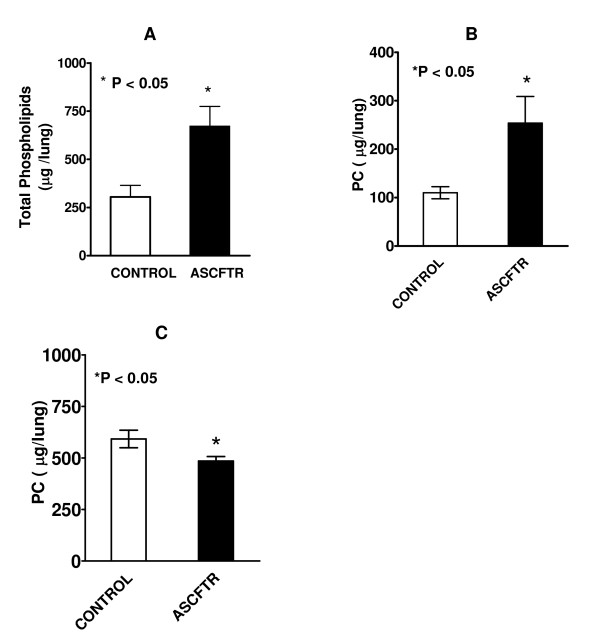
**Phospholipid and Phosphatidylcholine in large and small aggregates of alveolar surfactant**. Bronchoalveolar lavage fluid from control and ASCFTR animals was centrifuged to obtain the large and small aggregate fractions of alveolar surfactant. Lipid extracts were evaluated for total phospholipids **(A) **and phosphatidylcholine (PC) **(B)**. Results are mean ± SE of 7 animals in each group and are expressed as total μg lipid in lung. Total phospholipids and PC were higher in the ASCFTR rats. * P < 0.05. The mass of phospholipid was calculated by assuming that phospholipids phosphorous comprised 4% of the lipid mass. **(C) **The PC levels were lower in the small aggregate (SA) fraction in the ASCFTR rats in comparison to controls (* P < 0.05, n = 7, each).

Several studies have evaluated surfactant subfractions of BAL fluid samples to investigate the in vivo functions of ATII cells. The LA fraction represents the newly secreted surfactant which is capable of reducing the surface tension whereas the SA fraction contains old and inactive surfactant components [20-22]. The alveolar surfactant homeostasis is maintained by different mechanisms involving secretion and clearance as discussed above. The BAL fluid of ASCFTR-treated lungs showed higher total PL and PC contents in the LA fraction of lung surfactant (figures 2A and 2B). The increase in alveolar surfactant PC in LA fraction is possibly due to higher secretion, decreased conversion into SA fraction, or a combination of both. The SA fraction of BAL fluid showed decreased level of PC in the ASCFTR-treated group (figure 2C) suggesting decreased conversion of LA to SA fraction of lung surfactant. This was also confirmed by distribution analysis of total phospholipids and PC that showed decreased proportion of lipids in the SA fraction (Table 1). However, we cannot exclude the contribution of increased clearance of SA fraction to overall decrease in the PC pool in SA fraction of lung surfactant in ASCFTR rats.

**Table 1:** Percent distribution of phospholipids and phosphatidylcholine in the large and small aggregates of alveolar surfactant

	Control	ASCFTR	P value
Phospholipid			
Large Aggregates	25.7 ± 4.0%	48.6 ± 6.7%	<0.05
Small Aggregates	74.3 ± 4.0%	51.4 ± 6.7%	<0.05
			
Phosphatidylcholine			
Large Aggregates	16.2 ± 2.5%	33.2 ± 4.7%	>0.05
Small Aggregates	83.8 ± 2.5%	66.8 ± 4.7%	>0.05

Relative distribution of total phospholipids and phosphatidylcholine between the large and small aggregates of alveolar surfactant is expressed as percent distribution. Results are mean ± SE of 7 animals in the control and ASCFTR groups. Results are significantly different at P < 0.05.

#### Surfactant proteins

**Figure 3:**
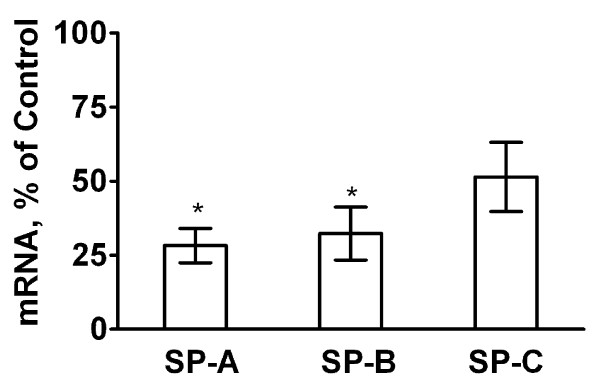
**Real-time PCR of surfactant-associated protein mRNA expression in lung**. Lung tissue was stored in the RNALater^® ^buffer for extraction of total RNA. Real-time PCR was performed for quantification of mRNA levels for SP-A, SP-B and SP-C and normalized to the house-keeping gene RPL13A. To obtain ratios of normalized mRNA levels for each protein for the ASCFTR and control lung, samples were paired with respect to the day of sacrifice. The average results of triplicate samples are paired and calculated as percent of control. Results are expressed as percent change (in comparison to the control) and are mean ± SE of 5 pairs of animals for each protein. * The difference was considered significant at ≥ 50% decrease.

Like surfactant lipids, surfactant proteins are also synthesized and secreted by ATII cells. Surfactant secretion and clearance is under regulatory control of surfactant proteins, particularly SP-A, since this most abundant surfactant protein plays a major role in surfactant homeostasis by promoting clearance and inhibiting secretion of lung surfactant [25-27,29]. The SP-A and SP-B mRNA levels in the lung tissue were lower in lungs of ASCFTR group compared to Control group (figure 3). However, western blot analysis of SP-A and SP-B in the LA fraction of alveolar surfactant and of the lung tissue did not show any difference between the two groups (not shown). The decrease in the mRNA levels of these two proteins in the ASCFTR group can possibly occur because of decreased ATII cell number in the lungs (see below) or altered cell function leading to changes in surfactant homeostasis. The surfactant proteins in the alveolar and lung compartments could remain unchanged because of corresponding decreases in the synthesis (as expected from decreased mRNA) and in the degradation of proteins. This probability would suggest dissociation between the synthesis and clearance processes for the surfactant protein and lipid components. We are unable to determine if such dissociation is due to immaturity or pathologic abnormality caused by a transient knock down of CFTR in the fetal lung.

**Figure 4:**
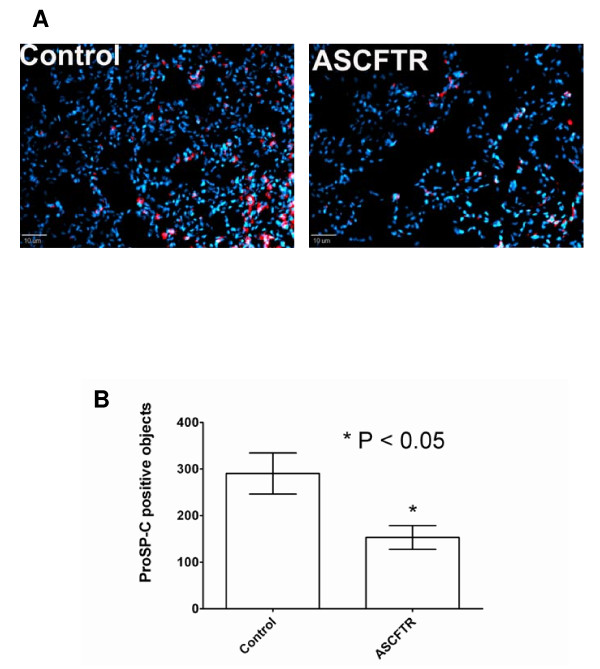
**Immuno-staining of lung sections for proSP-C**. **A**. Sections of frozen lungs from Control and ASCFTR rats were probed with polyclonal antibodies to proSP-C and then with Cy3-labeled secondary antibodies (red). The cell nuclei were counter-stained with DAPI (blue). The sections were viewed with a fluorescence microscope and photographed after deconvolusion and normalization of Cy3 fluorescence intensity to the same range of pixel density for the Control and ASCFTR specimens. **B**. A comparison of fluorescent object count between the Control and ASCFTR specimens showed a significant decrease in proSP-C positive cells (ATII cells) in the ASCFTR group in comparison to the Control group.

Although not significantly decreased, the SP-C mRNA in the ASCFTR group also showed a decreasing trend in comparison to the Control group. Since SP-C is a marker for ATII cells, this pattern for SP-C mRNA could be a reflection of decreased number of ATII cells. Immuno-staining of lung sections for proSP-C showed that fewer cells contained proSP-C in the ASCFTR lungs in comparison to the Control lungs (figure 4A). Analysis of five random fields in each group for fluorescent objects indicated that the number of proSP-C positive cells was significantly lower in the ASCFTR group in comparison to the controls (figure 4B).

### Studies in the ATII cells

#### PC Secretion

**Figure 5:**
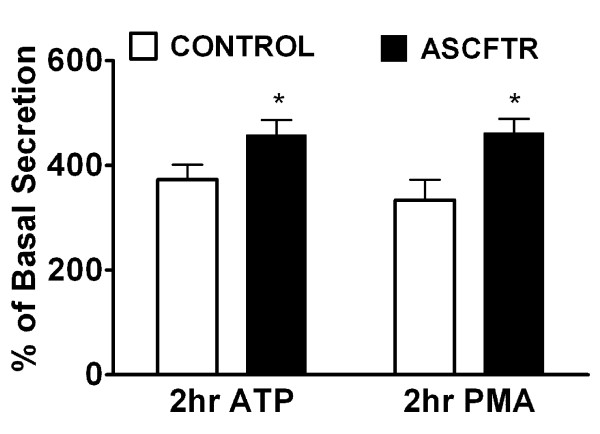
**Lung surfactant secretion in alveolar type II cells isolated from control and ASCFTR rats**. Alveolar type II cells were isolated from lungs of paired Control and ASCFTR rats. The cells were cultured for 20–22 h with [methyl-^3^H]choline to label cell phosphatidylcholine (PC). Following this culture period, adherent cells were incubated for 2 h without (basal secretion) or with addition of 1 mM ATP or 80 nM phorbol 12-Myristate 13-Acetate (PMA). The basal secretion was 1.05 ± 0.19% and 0.82 ± 0.11%, n = 6, in the control and ASCFTR cells, respectively (P > 0.05). The ATP and PMA-stimulated secretion is expressed as percent of the basal secretion. * P < 0.05 in comparison to the Control cells.

**Figure 6:**
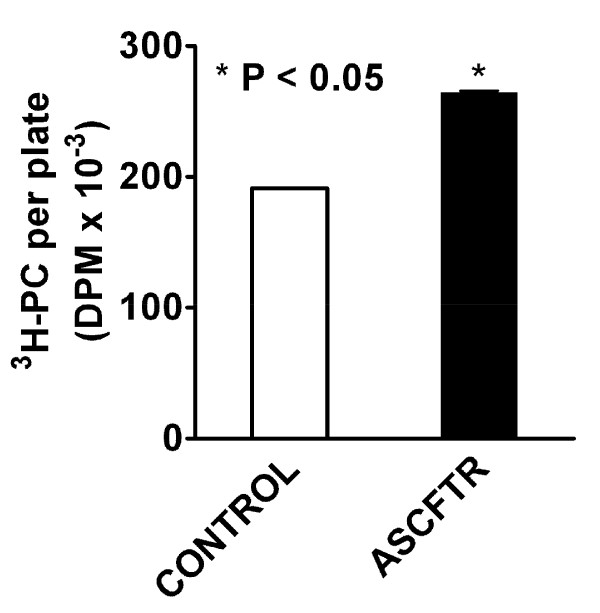
**Phosphatidylcholine pool in alveolar type II cells**. Equilibrium labeling of cell phosphatidylcholine (PC) with [methyl-^3^H]choline was used to measure the PC pool in alveolar type II cells. The cells were labeled for 20–22 h, as discussed for PC secretion studies. The radioactivity in the cell lipids was quantified. Results are mean ± SE of 6 separate experiments. The PC pool in ASCFTR cells was higher in comparison to the control cells. * P < 0.05.

The secretion of lung surfactant in ATII cells is a vital cell function, since its disruption would result in deficiency of alveolar surfactant and lead to respiratory distress. Therefore, evaluation of basal and stimulated secretion of surfactant PC can be used to test ATII cell function and establish phenotypic changes in the cells [23,43]. During the 2 h incubation, the basal PC secretion in ATII cells was similar in both groups (Control, 1.05 ± 0.19% and, ASCFTR, 0.82 ± 0.11%. n = 6 each, *P *> 0.05). The secretagogue-stimulated secretion was expressed as percent of basal secretion. The ATP-stimulated secretion was higher by about 30%. Similarly, the phorbol myristate acetate (PMA)-stimulated secretion was higher in the ASCFTR cells in comparison to the Control cells (figure 5). In order to determine if the secretion changes were accompanied by change in the cell PC pool, we measured equilibrium labeling (a measure of cell PC pool) of cells in these experiments. The labeling of cellular PC was higher by 30% in the ASCFTR cells when compared with the Control cells (figure 6). Since equivalent numbers of cells were plated for both groups, the results suggest higher PC mass and consequently higher absolute secretion of PC on per cell basis. Extrapolation of these findings to the in vivo function of ATII cells suggests that the increased pool of cell PC in the ASCFTR treated lungs could result in greater pool of PC in the alveolar surfactant.

#### Surfactant proteins

**Figure 7:**
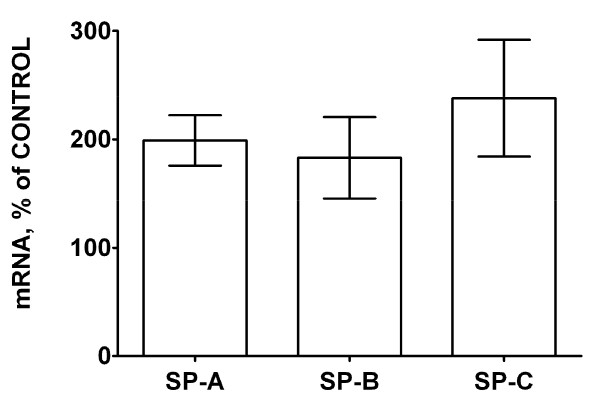
**Surfactant protein mRNA levels in alveolar type II cells**. Real-time PCR using total RNA from freshly isolated (uncultured) AT II cells was performed for quantification of mRNA levels of SP-A, SP-B and SP-C (n = 6, each). The results of triplicate analysis for each sample were paired between the control and ASCFTR samples and expressed as percentage of control. The mRNA levels (normalized to the house keeping gene RPL13A) for all three proteins were similar in the two groups. The results were considered significant at ≥ 200% (≥ 2-fold) of the Control.

Since evaluation of surfactant protein expression in lung tissue showed lower levels of SP-A and SP-B mRNA in the ASCFTR treated group (figure 3), we also measured the mRNA levels of these genes in the freshly isolated ATII cells (figure 7). For real-time RT-PCR analysis, control and ASCFTR cell RNA samples were paired for the day of cell preparation. The SP-A, SP-B and SP-C mRNA levels in freshly isolated ATII cells were similar in the two groups. Combining these results and the results from PC secretion experiments, our study suggests lack of coordination between the secretory function of surfactant phospholipids, the PC pool and the surfactant proteins mRNA levels suggesting altered ATII cell phenotype. The discrepancy in the surfactant proteins expression in the lung tissue (figure 3) and isolated ATII cells (figure 7), and the decreased immuno-staining for proSP-C in ASCFTR lungs (figure 4) suggest that lungs of ASCFTR rats contain fewer ATII cells in comparison to the Control lungs.

#### Calmodulin kinase and Calcium Regulating Genes

**Figure 8:**
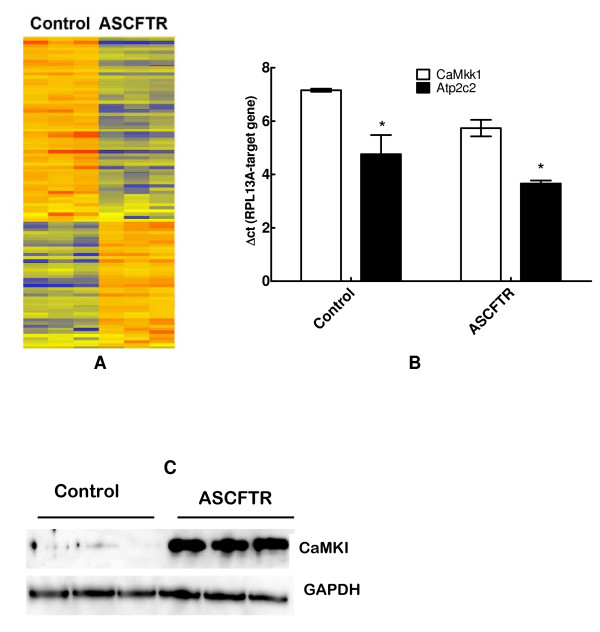
**Gene array analysis of ATII cell RNA**. Gene expression in adult type II cells isolated from animals that were infected *in utero *with adenovirus containing EGFP (Control) or ASCFTR constructs. **A**. Heat map for 101 genes in three separate preparations of type II cells demonstrating altered expression in the ASCFTR group in comparison to the Control group. **B**. Results of real-time PCR analysis of RNA from three separate preparations of freshly isolated type II cells showing enhanced expression of Ca^2+^-ATPase and calcium-dependent calmodulin kinase kinase1. Results (mean ± SE, n = 3) are expressed as change in threshold cycle for the target gene in comparison to the house keeping gene, RPL13A. A smaller ΔCt value indicates higher expression. * P < 0.05 in comparison to corresponding Control group. **C**. Western blot analysis showing elevated expression of calmodulin kinase I (upper panel) relative to house-keeping protein GAPDH (lower panel). The results of real-time PCR and Western blot analysis support the findings of Gene array analysis.

To obtain additional evidence for phenotypic changes in ATII cells, the total RNA from freshly isolated ATII cells was evaluated for altered gene expression by Microarray analysis using Affymetrix GeneChip^®^. Gene array analysis of 30,000 genes on the chip returned 4067 genes above the background level. The heat map demonstrating significantly altered expression of 101 genes in adult ATII cells as a result of transient *in utero *knock down of CFTR (P < 0.001 or better for all) is depicted in figure 8A. Forty genes showed at least 50% decrease and 22 genes showed greater than 2-fold increase in comparison to the Controls. These included Atp2c2 and CaMkk1 genes that regulate calcium transport or calcium related processes, respectively. Because ASCFTR cells demonstrated increased secretion of lung surfactant upon stimulation with secretagogues, we further verified altered expression of these two genes by real-time PCR. The expressions of CaMkk1 and Atp2c2 in the ASCFTR group were 281 ± 53% and 216 ± 14% of the Control group (figure 8B). CaMkk1 can phosphorylate CaMKI and increase its activity several folds [44]. Western blot analysis showed highly elevated expression of CaMKI (figure 8C). Since Ca^2+ ^is required for stimulated secretion of surfactant [24,45,46], increased expression of proteins regulating intracellular Ca^2+ ^levels and Ca^2+^-dependent protein kinase activity could be one possible mechanism for increased surfactant secretion. However, additional or alternate mechanisms like increased receptor density for purinergic receptors (for ATP effect) and/or increased PKC activity (for both ATP and PMA effects) could also play a role in increasing surfactant secretion.

## Discussion

The molecular basis of fetal lung development is believed to be under genetic and hormonal control [47,48]. Molecular cloning studies have shown that CFTR gene expression is regulated during lung development [49]. In addition, changes in CFTR expression can regulate fetal lung development [33]. We have previously shown that transient CFTR over-expression in the fetal lung following *in utero *gene transfer can modulate lung cell differentiation and maturation [13]. The *in utero *gene transfer is a useful tool to evaluate the developmental role of a gene in lung development [15,16]. Previous studies have demonstrated that the gene knock down after *in utero *transfection with antisense CFTR is transient [13,34]. The hypothesis is that this technique aims to alter gene expression in undifferentiated multipotent cells lining the developing airways. The infection efficiency or the cell types infected cannot be determined in these studies because of low virus dose resulting in low infectivity. Also, such determination would not be of significance because of the ongoing cell differentiation and proliferation during lung development. Although somewhat invasive, the fetuses treated with the control gene show normal differentiation and growth [14]. Our current study demonstrates that transient *in utero *knock down of CFTR with antisense constructs can alter the ATII cell phenotype in the adult animals suggesting persistent changes in cell phenotype possibly due to altered lung development.

The transient perturbations in gene expression due to ASCFTR treatment in the fetal lung were associated with altered surfactant status in the adult lung as suggested by increased alveolar pool size of surfactant lipids (figures 2A and 2B) without affecting the surfactant protein levels. Similar results have been reported for surfactant status in CF mice or patients [18,30,50]. Non-infected CF mice showed increased phospholipids pool in large aggregates without accompanying change in the SP-A levels [30]. Also, the alveolar surfactant phospholipid composition and function are altered in CF patients [18,30,50]. Our study suggests that the increase in large aggregates could result from higher surfactant secretion rather than decreased clearance of inactive components in the small aggregates (figure 2C). The size of alveolar surfactant pool is maintained by secretion and clearance processes which are coupled in normal lung [23,29]. The uptake of surfactant by ATII cells and by macrophages are the main routes of clearance and only a small portion of surfactant is cleared by upwards movement along the airways [23,29]. The increased pool size and increased secretion would suggest altered surfactant turnover in ASCFTR rat lungs. Previous studies also suggested altered ATII cell phenotype in mutant CFTR mice as they showed higher alveolar phospholipid because of the decreased uptake of alveolar PC [30,51]. Thus, two different approaches, transient (this study) and stable knockdown of CFTR [30], show alterations in ATII cell phenotype albeit in different processes. Although not directly related to surfactant metabolism, previous studies with isolated fibroblasts and platelets from CF patients also showed a change in PC metabolism since these cells showed increased PC synthesis [52]. The ASCFTR- treated ATII cells also show a larger cell PC pool (figure 6) and increased PC secretion that could contribute to increase alveolar pool of lung surfactant (figures 2A and 2B). The increase in PC secretion was observed with both ATP and PMA (figure 5) suggesting that the altered response may involve multiple pathways. One of these is likely the PKC-mediated pathway, since ATP stimulation of purinergic receptors [53,54] and consequent increases in intracellular Ca^2+ ^and diacylglycerol [28,55] would also stimulate PKC, while PMA directly stimulates PKC activity to increase surfactant secretion [24,28,55,56]. The increased expression of Atp2c2 and of Ca^2+^-dependent CaMkk1 and CaMKI (figure 8) would suggest that the Ca^2+^-dependent surfactant secretion [24,40,45,46] could be also elevated in ATII cells in the ASCFTR group. Although deficiency of surfactant proteins particularly SP-A could explain higher secretion and content of alveolar PC [25,29], its deficiency in lung or large aggregates was not observed in spite of decreased mRNA level in the lung tissue. Our study was not designed to determine the underlying mechanism of increased secretion which could occur through several processes including increased Ca^2+^-signaling, higher cell affinity to secretagogues because of increased receptor density, increased activity of intermediary steps like PKC activation for regulation of secretion, or more efficient fusion and exocytosis of lamellar body contents in the ASCFTR group.

The observations of decreased mRNA levels (figure 3) without accompanying decreases in SP-A and SP-B protein levels in the lung tissue of ASCFTR rats also suggest altered ATII cell phenotype. We cannot determine if dissociation between the transcription and translation processes contributed to this discrepancy. Because the mRNA levels of these proteins were not decreased in ATII cells (figure 7), it is likely that a decrease in the ATII cell number (figure 4) contributed to the overall decrease in surfactant protein mRNA in ASCFTR lungs. We suggest that the observed changes in ATII cell characteristics and cell number could occur because of developmental and cell differentiation-related changes. In previous study [13], developmental and differentiation related changes with CFTR over-expression possibly contributed to accelerated differentiation of fetal lung cells.

The results of transient perturbation in CFTR expression in the fetus suggest the importance of CFTR in fetal lung development [16,34]. The mechanisms that modify the ATII cell phenotype in the adult animal are likely complex but could be related to early effects of CFTR on expression of smooth muscle function-related genes and on mechanical stretch in the fetal lung [9-11]. Previous study has suggested that *in utero *over-expression of CFTR increases stretch in the fetal lung apparently through altered expression of myosin light chain kinase and myosin light chain phosphorylation [12]. Therefore, we presumed that decreased mechanical stretch results from CFTR knock down in the fetal lung that could possibly inhibit maturation of ATII cells [2,3,57]. Our current study of alterations in ATII cell function with transient knock down of CFTR gene expression further supports the modification of fetal lung development and differentiation processes by *in utero *gene delivery [13]. The resulting changes, like altered ATII cell physiology, apparently persist in the adult life of the animal suggesting that the effects of such interference in lung development in the fetus could linger for long periods. Such outcome supports the previously stated postulate that genetically encoded injury could have immediate effects even with apparently normal lung function and appearance [13]. Such a scenario can be seen in CF where the deleterious pathway begins early in the fetal lung [4]. However, several compensatory mechanisms may function in CF in an organ-specific manner, since the effects of global deficiency of CFTR manifest markedly differently in different organs. Although some of these mechanisms in the lung may be initiated during the period of *in utero *gene disruption in our protocol, extensive studies would need to be undertaken for such determination.

In summary, this study has shown that *in utero *transient disruption of CFTR gene results in lasting phenotypic change in ATII cell, altered surfactant homeostasis, and expression of several genes in adult rat ATII cells. The underlying mechanisms for change in ATII cell function is unclear but could result from diminished mechanical stretch in the fetal lung and result in delayed cellular differentiation. Further detailed investigations would be needed to establish if decreased number of ATII cells and metabolic abnormalities also contribute to altered lung surfactant homeostasis.

## Conclusion

Transient *In utero *disruption of CFTR can affect adult lung characteristics suggesting that events in fetal life can have persistent and long lasting effects in the adult life.

## Methods

### In utero gene transfer

All procedures involving animal experiments were approved by the Institutional Animal Care Committee. The *in utero *gene transfer technique is described previously [34]. It involves intra-amniotic administration of constructs containing the gene of interest. For this study, the adenovirus was constructed from an ATCC plasmid containing exons 1–6 of *cftr *cloned into a recombinant adenovirus in the antisense direction (AdCMVAScftr). Briefly, timed-pregnant Sprague Dawley rats at 16 days of gestation were anesthetized by inhalation of 5% isoflurane. Anesthesia was maintained by inhalation of 2% isoflurane. A laparotomy was performed, uterine horns were exposed, and adenoviral particle suspension in Dulbecco's Modified Essential Medium was injected into amniotic sac of each fetus using a fine gauge needle. The injected volume was estimated at 10% of the amniotic fluid volume and the final concentration of recombinant adenoviruses was 10^8 ^plaque-forming units (pfu)/ml [14]. Control animals were similarly injected with recombinant adenovirus containing enhanced green fluorescence protein (EGFP) reporter gene (generous gift of J. Kolls, University of Pittsburgh). We did not use sense-CFTR in the controls because our previous studies demonstrated that CFTR over-expression, as would be achieved with the sense-CFTR, caused morphologic changes in the developing lung [13]. Because of transient expression of genes, we injected all littermate fetuses on the same day with either the control or the ASCFTR construct in separate pregnant animals. All rats were housed in standard housing conditions in unfiltered cages until normal delivery at term. The newborn pups were weaned at 21 days of post natal life and maintained under normal housing conditions.

### Type II cell isolation

At 3–4 months of age, adult male rats were used for isolation of alveolar ATII cells. We used the previously published protocol [58] as modified by Sen and Chander [59]. In brief, rats were anesthetized with sodium pentobarbital (50 mg/kg, intraperitoneal), tracheostomized, and exsanguinated. The lungs were ventilated and cleared of residual blood by perfusion through the pulmonary artery. The lungs were removed, lavaged, and treated (three times) with intratracheally instilled elastase solution (3 U/ml) (Worthington, Biochemicals, Freehold, NJ). The lung lobes were minced on a tissue chopper, suspended in buffer containing DNase, and mixed vigorously for 2 min in a water bath at 37°C. The free cells in suspension were sequentially filtered through nylon filters of decreasing pore size, and centrifuged for 10 min at 300 × g. Cells in the pellet were suspended in minimum essential medium (MEM) and incubated for 1 h at 37°C on IgG-coated bacteriologic culture dishes. The free cells were subsequently collected by "panning," centrifuged and suspended in MEM containing 10% fetal bovine serum (FBS). Acridine orange staining [60] showed that ATII cells accounted for ~ 90% of the total cells. Aliquots of the isolated cells were saved for RNA studies and remaining cells were plated on 35-mm tissue culture plates at a density of 1.5 × 10^6 ^cells in 1.5 ml MEM-10% FBS. Plated cells were incubated for 20–22 h at 37°C in humidified air containing 5% CO_2_. Cells attached to the tissue culture plates after overnight incubation were >92% ATII cells as determined by acridine orange staining.

### PC Secretion and Equilibrium Labeling of Cell PC

For studies on PC secretion, isolated cells were plated in MEM containing 10% FBS and 0.5 μCi of [methyl-^3^H] choline (Amersham, Arlington Heights, IL) and cultured for 20–22 h. Following this culture period, the adherent cells were extensively washed to remove non-adherent cells, serum proteins and unincorporated radioactivity. One ml of MEM was added to each plate. Duplicates were done for each rat. After incubation in 5% CO_2 _at 37°C for 30 min (equilibration period), one set of plates was removed and labeled as 'zero' time secretion. To the other plates 10 μl of ATP or PMA was added to yield final concentrations of 1 mM or 80 nM, respectively. Control plates (basal secretion) received 10 μl of water. The plates were returned to the incubator for 2 hr. Following incubation, the media were removed and centrifuged (300 × g for 10 min at 4°C) to pellet any cells that might have detached during incubation. These cells were later pooled with those recovered from the plates. Lipids from both media and cells were extracted (see below) after addition of [methyl-^14^C] dipalmitoyl phosphatidylcholine (DPPC) (Amersham) and egg PC (Sigma Chemical Co.) as the carrier lipid. Radioactivity in lipid extracts was measured in a liquid scintillation counter. The recovery of [^3^H]-choline- labeled phospholipids was corrected using the recovery of ^14^C-DPPC. Phospholipid secretion was calculated as percent secretion = (DPM in medium lipids × 100)/(DPM in lipids in medium plus cells). For quantification of PC mass, ATII cells were incubated with [^3^H]choline for 20–22 h and the radioactivity in cell PC was measured after lipid extraction. Previous studies have shown that the incorporation of [^3^H]choline achieves plateau by 16 h of incubation and does not change thereafter in continued presence of [^3^H]choline [59]. At these equilibrium labeling conditions, the radioactivity in cell PC is representative of cell PC mass.

### Lipid extraction and analysis

Lipids were extracted from all samples using chloroform-methanol system [61]. The extracted lipids were dissolved in chloroform-methanol (20:1, v/v). Total phospholipids in the lipid extract were determined by analysis of phospholipid phosphorus (PLPi) following digestion of lipids in perchloric acid (70–72%) to release inorganic phosphorus (Pi) that was measured according to previously published methods [62,63]. Lipids extracts were analyzed for PC after separation of phospholipids by thin layer chromatography (TLC) [64]. Lipids were visualized by exposing the TLC plates to iodine vapors, and PC was identified by co-migration of authentic phospholipid standards [62,63]. The spots containing PC were removed from the plates and directly processed for digestion of lipids in perchloric acid (see above) and for spectrophotometric determination of the phosphorus content [62].

### RNA Extraction and Analyses

Total RNA from samples of freshly isolated ATII cells and or samples of lung tissue from different cohorts of animals were extracted using the RNeasy kit (Qiagen, Inc). The yield and purity of the isolated RNA were determined by A_260 _and A_280_. Gene array analyses of RNA samples from three separate preparations of freshly isolated ATII cells were performed at the DNA Microarray Facility at the Stony Brook University using GeneChip^® ^Rat Genome 230 2.0 array (Affymetrix).

### Real-time PCR

The expression of several proteins was measured by quantifying the mRNA levels by real-time PCR. In this study, we measured the mRNA levels for surfactant proteins A, B, and C and for Ca^2+^-ATPase and CaMkk1 relative to that of house-keeping gene (RPL_13a_) encoding for ribosomal protein L13A, which is a structural component of 60S ribosomal subunit. In our previous studies, we had observed large variations in the expression of GAPDH during fetal lung development, while RPL13A was more stable. The RT-PCR primer sets for the target and housekeeping genes (RPL_13a_) were obtained from commercial source (Superarray, Inc, Fredrick, MD). Triplicates of a 20 μl reaction mixtures containing 20 ng of RNA were analyzed for each surfactant protein and averaged. The comparative threshold cycle (C_t_) method, also known as the C_t _method [65], was used to calculate changes in mRNA levels according to the following equation.

ΔΔCt = ΔCt_experimental _- ΔCt_control_

Sample Difference = 2^-ΔΔCt^

Where, ΔCt_experimental _is the Ct value for experimental sample for the target gene normalized to the endogenous housekeeping gene and ΔCt_control _is the Ct value for the control sample for the same target gene also normalized to the endogenous housekeeping gene [65]. Control and experimental animals sacrificed on the same day were paired for comparison. Results from indicated groups of control and experimental animals are expressed as mean ± SEM of indicated number of such pairs.

### Protein Analysis

Total proteins were extracted from lung tissue samples using ~ 700 μl of fresh whole cell lysis buffer (PMSF 0.25 mM, EGTA 2 mM, 2 × LSB, protease inhibitor cocktail (Sigma, St Louis, Mo), 50 mM NaF and phosphatase cocktail solutions I and II (Calbiochem, San Diego, CA)). Samples were sonicated, boiled for 5 min and centrifuged to obtain soluble proteins in the supernatant fraction for measurement of total proteins. Protein concentrations were determined spectrophotometrically using a bovine-γ-globulin as standard [66]. Chemiluminiscence detection [67] was used to detect indicated antigen (SP-A, SP-B and CaMKI) by Western blot analysis using respective primary antibodies. The rabbit SP-B antibody was raised against Survanta® (Abbott Laboratories, Abbott Park, IL). This antibody recognized the precursor and the mature forms of SP-B in lung tissue and only the mature form in the LA fraction of alveolar surfactant. The SP-A antibody was obtained from commercial source (Chemicon International, Temecula, CA). The antibodies for CaMKI were obtained from Santa Cruz Biotechnology (Santa Cruz, CA). All primary antibodies were used at a dilution of 1:1000 and appropriate secondary antibodies were used at a dilution of 1:10,000 or 1:20,000. Equal amounts of total proteins (or volumes, in case of suspension of LA fraction) were loaded for each sample and the relative contents of target proteins were quantified after photo-imaging of Western blots (Hitachi Genetics Systems, Alameda, CA). Images were acquired by GeneSnap program (Syngene, Frederick, MD)

### Histochemistry and Immunocytochemistry

Lungs were fixed in methanol-free 4% buffered paraformaldehyde, mounted in paraffin, and thin (8 μm) sections were prepared. Slides were immersed in Histol-Clear solution (National Diagnostics, Atlanta, GA) to remove residual paraffin and then rehydrated through a series of washes with decreasing concentrations of ethanol. After rinsing in distilled water, slides were stained with hematoxylin and eosin (Fisher Scientific Inc) and examined by standard light microscopy. For immuno-staining for proSP-C, frozen lung sections were blocked for 1 h with donkey serum, incubated for 75 min with rabbit antibodies to proSP-C (kind gift of Dr. Jeffery A Whitsett, University of Cincinnati) at 1:500 dilution, washed, and incubated for 30 min with Cy3-conjugated secondary antibodies (donkey anti-rabbit, Santa Cruz Biotechnology) at 1:500 dilution. The sections were counter-stained with DAPI and viewed and photographed after deconvolution and normalization of fluorescence to the same pixel density range for the Control and ASCFTR specimens. Image software SLIDEBOOK was used to capture images and quantify fluorescence for object count. Five random fields in the peripheral lung sections were evaluated for fluorescence and compared between the two groups.

### Statistical Methods

All statistics were performed using GraphPad Prism software program (GraphPad, San Diego, CA). Unpaired student's *t *test was used to analyze data which were expressed as the mean ± SEM. Differences were considered significant at *p *< 0.05.

## Abbreviations

ATII: alveolar type II; Atp2c2: Ca^2+^-transporting ATPase; BAL: bronchoalveolar lavage fluid; CaMkk1: calcium-dependent calmodulin kinase kinase 1; CaMKI: calmodulin kinase I; CF: Cystic fibrosis; CFTR: Cystic Fibrosis Transmembrane Conductance Regulator; ASCFTR: antisense-CFTR; Ct: threshold cycle; DPM: disintegrations per minute; FBS: fetal bovine serum; LA: large aggregates; PCR: Polymerase chain reaction; Pi: inorganic phosphorus; PKC: protein kinase C; PL: phospholipids; PC: phosphatidylcholine; DPPC: dipalmitoyl PC; PMA: phorbol myristate acetate; SA: Small aggregates; SP: Surfactant protein

## Authors' contributions

AG and JH carried out *in utero *injections. AG and DC conducted biochemical analysis of lung surfactant, preparation of type II cells and surfactant secretion studies. AG, DC and EK conducted all RT-PCR studies and EK was responsible for immunostaining and analysis of lung sections. AG and EK conducted Western blot analysis of all samples. MD prepared RNA for Microarray analysis and analyzed results of Microarray data. JCC, JEL, and AC were responsible for overall supervision of studies, training of personnel for *In utero *treatment, surfactant biology, research planning and interpretation of results, and in continuous editing and revision of this manuscript written by AG. All authors reviewed this manuscript.
